# Surgical adhesive glue to repair first-degree perineal tears in vaginal birth: A randomised controlled clinical trial

**DOI:** 10.1016/j.ijnsa.2023.100130

**Published:** 2023-05-12

**Authors:** Thaís Trevisan Teixeira, Maria Luiza Riesco

**Affiliations:** a School of Nursing, University of São Paulo. Midwife in clinical practice at Casa Angela Birth Center, Brazil; b School of Nursing, University of São Paulo, Brazil

**Keywords:** Parturition, Perineum, Lacerations, Tissue adhesives, Cyanoacrylates, Midwifery, Clinical trial

## Abstract

**Background:**

Perineal tears in vaginal birth are highly prevalent and may be related to physical and psychological trauma. Surgical glues are an alternative repair method to avoid the pain that may be caused by perineal repairs with sutures.

**Objective:**

To evaluate the effectiveness of surgical adhesive glue in reducing perineal pain when compared to sutures in first-degree perineal tears resulting from vaginal birth.

**Design:**

Open-label parallel-group randomised controlled trial.

**Setting:**

An alongside birth centre in Sao Paulo, Brazil.

**Participants:**

84 intrapartum women with first-degree perineal tears needing repair.

**Methods:**

In the experimental group (*n* = 42), the perineal tears were repaired with Epiglu® surgical glue (ethyl-2-cyanoacrylate); in the control group (*n* = 42), the tears were repaired with Vicryl Rapide® (polyglactin 910) sutures. The primary outcome was the intensity of perineal pain after birth measured by a numeric pain rating scale ranging from 0 to 10 points. The secondary outcomes were healing, measured by the “Redness, Oedema, Ecchymosis, Discharge, and Approximation" scale; women's satisfaction with the perineal repair, measured by a visual analogue scale; and the time necessary to complete the repair. Data were collected during postpartum hospitalisation and 10–20 days after discharge, from December 2020 to May 2021. Data were analysed using bivariate analysis and linear models by intention-to-treat.

**Results:**

36–48 h after birth, the mean of perineal pain was 0.2 (95% Confidence Interval [CI] 0.1–0.8) in the experimental group and 0.9 (95% CI 0.5–1.5) in the control group; the perineal healing score was 0.7 (95% CI 0.4–1.2) and 0.8 (95% CI 0.5–1.2), in the experimental and control groups, respectively; satisfaction was higher among women in the experimental group (88.1% versus 83.3% in the control group). After discharge, the mean of perineal pain was 0.1 (95% CI 0.0–0.5) in the experimental group and 1.4 (95% CI 0.8–2.2) in the control group; the perineal healing score was 0.0 (95% CI 0) and 1.0 (95% CI 0.7–1.3) in the experimental and control groups, respectively. Satisfaction was higher in the experimental group (94.9% versus 75.0%). The longitudinal analysis showed statistically significant differences between the groups regarding perineal pain and women's satisfaction. The average time necessary for perineal repair was 6.0 (95% CI 4.7–8.7) minutes in the experimental group and 9.7 (95% CI 8.3–11.5) in the control group (*p* < 0.001).

**Conclusions:**

Surgical glue resulted in less perineal pain, faster repair, and greater satisfaction than perineal sutures after birth. The healing process was similar in both cases.

**Tweetable abstract:**

Surgical glue was less painful and promoted greater satisfaction after birth compared to sutures in women with first-degree perineal tears.

**Registration:**

Registered on The Brazilian Clinical Trials Registry number RBR-52y5tq (http://www.ensaiosclinicos.gov.br/rg/RBR-52y5tq/), on July 16, 2020. The first recruitment was on December 17, 2020.

**What is already known**

• Perineal tears have a high prevalence and are currently performed with sutures, associated with pain during repairing and in the days following the procedure.
• Surgical adhesive glue appears to be an alternative to suture repair, but the benefits of different types of surgical adhesive glue are still unclear.

**What this paper adds**

• This clinical trial found that surgical glue (ethyl-2-cyanoacrylate) reduced pain and repair time and increased women's satisfaction compared to suturing.
• Slow application of the glue, drop by drop, facilitated the repair.

## 1 Background

Perineal trauma is defined as any discontinuity of perineal tissues during vaginal birth (Frolich and Kettle, 2015). It can occur spontaneously or be caused by a professional through an episiotomy, defined as a surgical incision with scissors or a scalpel performed in the perineal region during birth (Jiang et al., 2017).

The classification currently adopted for spontaneous perineal trauma comes from the late 1990s and divides it into four degrees. First-degree trauma or tear affects only the skin and mucosa; second-degree tear affects perineal muscles (except for the anal sphincter); third-degree tear affects the anal sphincter and is subdivided into 3a when reaching up to 50% of the external anal sphincter, 3b when affecting more than 50% of the external anal sphincter, and 3c when also reaching the internal anal sphincter. When a tear affects the rectal epithelium, it is considered fourth-degree (Sultan, 1999).

Perineal trauma is an outcome that occurs in most vaginal deliveries. It affects primiparous more often than multiparous women (Leal et al., 2014; Abedzadeh-Kalahroudi et al., 2019; Smith et al., 2013). In England, around 90% of primiparous and 69% of multiparous women had perineal trauma during birth (Smith et al., 2013). Studies in Sweden, Iran, and Spain showed an incidence of 66%, 83%, and 91%, respectively (Abedzadeh-Kalahroudi et al., 2019; Jansson et al., 2020; Mora-Hervás et al., 2015).

Brazil follows the same patterns, with a range of 56% to 79% of perineal trauma incidences in vaginal deliveries (Leal et al., 2014; Leal M do et al., 2014; Lins et al., 2019). Despite this high global incidence, first-degree trauma is the most common and usually does not require suturing, except in cases of excessive bleeding or local anatomical distortion (Leal et al., 2014; Lins et al., 2019; Lopes et al., 2019; Brasil. Ministério da Saúde 2017; National Institute for Health and Clinical Excellence 2017; American College of Obstetricians and Gynecologists' Committee on Practice Bulletins 2016).

Currently, the Brazilian national guideline for normal birth care (Brasil. Ministério da Saúde 2017) instructs that women should be advised to have a suture for first-degree tears to improve healing unless the edges are well-attached; therefore, a portion of these perineal traumas will need to be repaired. Regarding second-degree tears and episiotomy, the muscle should be sutured to improve healing, and if the skin is well attached after this step, it does not need suturing. Such recommendations are in line with those of the United Kingdom's National Institute for Health and Care Excellence (National Institute for Health and Clinical Excellence 2017).

One of the factors that can undermine healing and potentially increase painful sensations is the high tension of the suture and the thread acting as a foreign body in the trauma site, which may be associated with repair using surgical thread (Childs and Murthy, 2017; Guo and DiPietro, 2010). There is a possible improvement in well-being when suturing is not used to repair perineal trauma (Elharmeel et al., 2011).

Studies have addressed the use of cyanoacrylate-based surgical glue as an alternative to suturing in women with first-degree perineal tears that needed repair. The Queensland Clinical Guidelines incorporate its use as one of the techniques to be considered in the case of first-degree perineal tears (Queensland Clinical, 2020).

Surgical glue is presented as one of the alternatives to traditional methods of surgical repair. Its use in medicine began in 1959 with successive improvements in search of efficacy and safety in its use (Coover et al., 1959; Bhaskar and Frisch, 1968).

Cyanoacrylates have been studied in several areas of health, such as dentistry and general surgery (Adoni and Anteby, 1991; Chow et al., 2010; Borie et al., 2019). But the first use in obstetrics was reported in 1991 when catgut and Histoacryl® surgical glue were compared for the repair of the skin layer in episiotomies, and lower pain scores were found with the use of surgical glue (Adoni and Anteby, 1991).

The cyanoacrylate monomer is a low-density liquid polymerising in long chains with ions and hydroxyl, such as blood and body fluids, forming a thin layer that causes tissue adhesion and has had its antimicrobial properties confirmed in vitro (Hollander and Singer, 1998; Bhende et al., 2002).

Surgical glue seems to be a viable and safe alternative when perineal trauma repair is necessary (Queensland Clinical, 2020; Marks et al., 2020; Feigenberg et al., 2014). However, according to the existing literature, not all types of cyanoacrylates have been explored, especially the lower-cost ones. In addition, more well-designed studies are needed to analyse the effectiveness and superiority of surgical glues concerning suturing perineal trauma in childbirth (Elharmeel et al., 2011; Feigenberg et al., 2014).

This study aimed to evaluate the effectiveness of surgical adhesive glue in reducing perineal pain in the repairing of first-degree perineal tears resulting from vaginal birth when compared to suturing. A lowest-cost glue approved to be used on skin and mucous membranes was tested.

## 2 Methods

### 2.1 Study design

An open-label parallel-group randomised controlled trial.

This study was registered on The Brazilian Clinical Trials Registry number RBR-52y5tq (http://www.ensaiosclinicos.gov.br/rg/RBR-52y5tq/) on July 16, 2020. The first recruitment was on December 17, 2020.

### 2.2 Setting

The study was carried out at an alongside birth centre in a hospital in the south region of São Paulo city, Brazil.

It performs an average of 370 births per month, of which approximately 70% are vaginal. The facility has five delivery rooms and 48 rooming-in beds.

A team of midwives provides care for normal births, and obstetricians are called upon in case of pathologies. The birth centre receives midwifery and medical students for training. Women who need pharmacological analgesia or anaesthesia are transferred from the birth centre to the operating room, located in the same hospital but in another sector.

### 2.3 Participants and sample

The population consisted of women admitted for birth at the birth centre. All women aged 16 years or older, with cervical dilatation of up to 6 cm, able to communicate effectively at the time of invitation to participate in the study, and who agreed to undergo perineal repair with surgical glue or suture were considered eligible, regardless of parity, foetal position, or number of foetuses. They did not use oral steroid medication and did not have leukorrhea or any sign of perineal infection, diagnosis of diabetes mellitus or gestational diabetes, history of allergy to surgical adhesive glue or formaldehyde, current diagnosis of COVID-19, or difficulty in understanding the Portuguese language.

Women were invited to participate in the study during hospitalisation, where the consent form was presented, read, accepted or refused, and signed if accepted. Inviting them during prenatal care was impossible, as the follow-up was carried out in different settings. The first author (TTT) was responsible for inviting the women to participate in the study.

For consistency, the repairs with surgical glue were performed by three midwives who were previously trained to make them. The suture repairs were completed by midwives who were part of the birth centre's staff. Students did not perform any perineal repairs.

The inclusion criterion was the occurrence of a first-degree perineal tear that required repairing according to the healthcare professional assisting the birth. Exclusion criteria were episiotomy or second, third, or fourth-degree tear associated or not with a first-degree tear, intra-amniotic infections, vulvar varicosities, or complications arising from birth or postpartum requiring transfer to the intensive care unit.

Previously, a pilot study was carried out with 20 women to determine the feasibility of this randomised controlled trial, observing a Cohen's effect size of 0.5129 in the longitudinal comparison of perineal pain means (Teixeira, 2018). For this interaction effect to be detectable in an ANOVA model for repeated measures with a type I error of 5% and test power of 95%, it was necessary to include at least 70 participants, 35 in the experimental group and 35 in the control group. The G Power 3.1.9.7 programme was used to calculate this sample.

A loss of 20% was estimated in the calculated sample; therefore, 14 participants were added, increasing the total to 84. The women in the sample were distributed to the experimental group (*n* = 42) and control group (*n* = 42). The experimental group consisted of women who underwent perineal repair of lacerations with ethyl-2-cyanoacrylate surgical glue under the trade name Epiglu®. The control group consisted of women who underwent perineal repair by suturing with polyglactin 910 thread under the trade name Vicryl Rapide®.

### 2.4 Randomisation

The allocation of women to the two study groups was done randomly through a sequence of simple computer-generated random numbers. The numbers were sealed into brown, opaque, and sequentially numbered envelopes by a research assistant not participating in the data collection and analysis.

The envelopes were placed in a password-protected box and stored at the birth centre's office. Then, after confirming that the participant met the inclusion criteria, the professional responsible for the data collection accessed and opened the sealed envelope, following the pre-established numerical sequence and allocating the participant to the indicated group. The researcher, the research assistants, and any professionals involved in the data collection had no access to the randomisation sequence before the envelope was opened.

Given the nature of the interventions and outcomes, there was no possible blinding of the trial. The women and the researcher were aware of the type of perineal repair performed, and, during the healing assessment, fragments of the material used in repairing might have been visible, distinguishing the surgical adhesive glue or the suture.

### 2.5 Interventions

#### 2.5.1 Surgical adhesive glue repair

Surgical glue with ethyl-2-cyanoacrylate is indicated for external use in surgical incisions and recent lacerations, mucous membranes, or mucocutaneous junctions (buccal and vaginal cavities) in the postoperative period. It is used as a substitute for sutures in lesions and has a bacteriostatic, sealing, and waterproof effect.

The Epiglu® adhesive is produced by the German company Meyer-Haake, registered by the National Health Surveillance Agency of Brazil-Anvisa. It is available in the Epiglu® multidose presentation, one vial with 3 ml; the vial's Epiglu multidose presentation allows adhesion of 20 lesions of up to 10 cm in length. Its liquid polymerises quickly in contact with water and body fluids; therefore, minimal amounts are enough to ensure adhesion to the treated tissue.

The product must be stored at −18 °C, and its shelf life is 24 months; if stored in a refrigerator, the shelf life is reduced to 6 months. The manufacturer recommends transporting the product between 15 °C and 30 °C for a maximum of 72 h.

In the experimental group, the repair was performed using the following technique:

1 Evaluate the condition of the perineum and confirm the degree of laceration reported by the health professional attending the birth.
2 If necessary, insert a gauze pad into the vaginal introitus to prevent blood from spilling and to keep the perineal trauma area drier.
3 Dry the area where the perineal repair will be performed with gauze.
4 Aspirate the glue with the sterile *Pasteur* pipette.
5 Approximate the edges of the laceration with the thumb and index finger.
6 Hold the sterile *Pasteur* pipette, squeezing it lightly while applying one drop of Epiglu®.
7 Allow the drop to run over the approximated edges. If necessary, spread the solution using the pipette's tip to make the glue layer as thin as possible.
8 Check if the repair is adequate and apply more surgical glue in the regions where the approximation is inadequate. Make sure to apply the least amount of glue possible to promote better adhesion.

#### 2.5.2 Suture repair

In the control group, the fast-absorbing 3–0 and 2–0 polyglactin 910 thread was used with the following technique:

1 Evaluate the condition of the perineum and confirm the degree of laceration reported by the health professional attending the birth.
2 If necessary, insert a gauze pad into the vaginal introitus to prevent blood from spilling and to keep the perineal trauma area drier.
3 Dry the area where the perineal repair will be performed with gauze.
4 Inject local anaesthesia with 1% lidocaine without vasoconstrictor.
5 Suture with Vicryl Rapide® thread using continuous stitches without anchoring.
6 Check if the perineal repair is adequate.

The research team (first author and two birth centre midwives) confirmed the degree of laceration and performed the repair with surgical glue. The suture repair was performed by the same professional who attended the birth. The birth centre team was trained to practice the suturing technique adopted in the control group according to the institutional protocol.

### 2.6 Outcomes

The primary outcome was the intensity of perineal pain after birth. The secondary outcomes were healing, women's satisfaction with the perineal repair, and time necessary to complete perineal repair.

Pain was assessed through a numeric pain rating scale from 0 to 10, where 0 represented no pain, and 10 was the worst possible pain.

Healing was evaluated using the “Redness, Oedema, Ecchymosis, Discharge, and Approximation" scale (REEDA) (Hill, 1990). To evaluate these parameters, a specific ruler for assessing perineal trauma, the Peri-Rule® (Alison et al., 2002), was used, sanitised with water, soap, and 70% alcohol after each use, and then wrapped in a plastic film (that was discarded after use), to protect the ruler. The five items were scored from 0 to 3, which could reach a maximum of 15 points on the scale. The maximum score of 15 corresponded to the worst healing of the perineum.

For the analysis of satisfaction, a visual analogue scale was created during the pilot study and presented to the research participants on a 5 cm x 20 cm chart, showing four faces with different features representing satisfaction with the method used, being attributed to the characteristics of "very dissatisfied", "dissatisfied", "satisfied" or "very satisfied", following the direction of the faces from left to right.

The time was recorded with the aid of a previously existing digital stopwatch mounted on the wall of the delivery room. The time was counted beginning after the drying of the perineal repair area, described in both techniques, until the professional indicated the completion of the repair.

### 2.7 Implementation

Data were collected at four different stages.

• **Stage 1:** inclusion of women in the study, allocation to one of the groups, evaluation of the perineal laceration, completion of the repair, the timing of repair, and evaluation of outcomes at the time of perineal repair. Women were interviewed about pain and satisfaction with the repair up to 2 h after birth. Data were obtained by consulting the medical records, interviewing the women, and performing perineal examinations.

The following stages corresponded to the follow-up of women throughout the study in the postpartum periods described below.

• **Stage 2:** interview and perineal examination during hospitalisation between 12 and 24 h after birth.
• **Stage 3:** interview and perineal examination during hospitalisation between 36 and 48 h after birth. At this stage, the date of the next stage was scheduled.
• **Stage 4:** interview and perineal examination at the hospital between 10 and 20 days after birth. The researcher called the participant 2 days before the scheduled date to confirm attendance.

Data collection took place during the COVID-19 pandemic, respecting current health protocols. The hospital was one of the referral treatment units for COVID-19 cases in the city of São Paulo. Only a few hospitals were classified as referral units for treatment. The hospital where this research was conducted was one of them. Due to the increase in COVID-19 cases amongst the population of Sao Paulo city, the hospital's administration decided that postpartum women and newborns would not return for face-to-face evaluation at the hospital. After this decision, the evaluation of pain and satisfaction for Stage 4 (10 – 20 days after birth) was conducted via telephone calls for some of the participants. Nevertheless, they were aware of the assessment scales that had already been used in the previous stages during their interviews at the birth centre. To assess healing, participants were instructed to have the perineum evaluated during regular postpartum follow-up appointments with a doctor or nurse from the public health network.

### 2.8 Statistical methods

Double data entry was used in Excel software. For the descriptive analysis of numerical variables, measures of central tendency and dispersion were calculated: mean, standard deviation, and maximum and minimum values. The categorical variables were analysed by absolute and relative frequencies.

The time of perineal repair was compared between groups using Wilcoxon-Mann-Whitney for bivariate analysis. The longitudinal measures were compared with a linear mixed-effects model for the numerical variables and a generalised linear model for the cumulative binomial family for the ordinal variable.

Data were analysed by intention-to-treat. Results were considered statistically significant at alpha <0.05, and 95% confidence intervals (CI) were presented. All analyses were performed in the R 4.1.1 statistical package.

### 2.9 Ethics approval

The study was approved by the Research Ethics Committees of the School of Nursing of the University of São Paulo with approval number 3.890.595 on February 28, 2020, by the Instituto Israelita Albert Einstein (administrators of the Municipal Hospital) with approval number 3.957.461 on April 6, 2020 and by the Municipal Health Department of Sao Paulo with approval number 4.191.287 on August 4, 2020.

Women's participation was voluntary and followed all the determinations of Resolution n. 466 of December 12, 2012 of the Brazilian National Health Council, ensuring the protection of the rights of all involved in the research. All participants signed a consent form to participate in this study.

## 3 Results

Participants were included in the study from December 17th, 2020 to May 12th, 2021, and postpartum follow-up took place until May 28th, 2021.

During that period, 455 women were evaluated for eligibility, of which 371 were excluded. The reasons were: not meeting inclusion criteria due to the diagnosis of gestational diabetes, hospitalisation in advanced labour, being younger than 16 years old, and not understanding the Portuguese language well (*n* = 80). Among eligible women, six refused to participate, 285 were followed during labour and birth but were excluded because they did not have a first-degree tear that needed repairing, were discharged before birth, or gave birth outside of the period when one of the researchers was present at the birth centre.

Of the eligible women who had vaginal births, 36.3% (*n* = 86) had first-degree tears that needed repairing, 39.2% (*n* = 93) did not have tears, 5.1% (*n* = 12) had first-degree tears without the need for suture; 13.1% (*n* = 31) had second-degree tears, 0.4% (*n* = 1) had a third-degree tear, and 5.9% (*n* = 14) had episiotomies.

Therefore, 84 women with first-degree tears requiring repair were included in the study, while two other eligible ones could not be included as the birth occurred concomitantly with others, and the researcher could not be present at both. Forty-two were allocated to each group strictly following the pre-defined randomised sequence. All 42 women allocated to the control group received the pre-established intervention (suture); however, six women randomised to the experimental group did not receive the pre-established intervention due to failure in the adhesion of the surgical glue (the glue did not maintain adhesion to the tissue) and had a repair done with suture. Ultimately, 36 women received the experimental group intervention. Four women did not respond to the contact for the 10–20 days follow-up.

The groups were analysed according to the initial randomisation by intention-to-treat; therefore, the six experimental group participants who did not receive the surgical glue intervention remained in the experimental group for analysis.

**Fig. 1:**
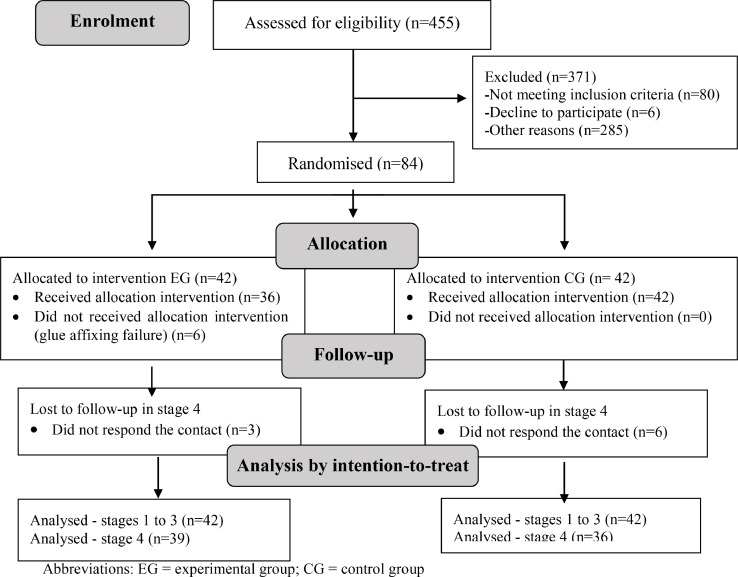
Data collection flowchart.

The following flowchart shows the data collection described (Fig. 1).

The table in supplementary material (file 1) presents the distribution of the characteristics of the experimental group and control group participants. The variables considered for characterisation of the participants were skin colour, education level, occupation, marital status, previously sutured perineal trauma, presence of a companion during birth, use of intrapartum antibiotic, age, gestational age, body mass index, previous birth, time between admission and birth, time between amniorrhexis and birth, and newborn weight.

Table 1 shows the averages of perineal pain evaluated through the numeric pain rating scale from 0 to 10 throughout the four stages of the study.

**Table 1:** Perineal pain score.

Stage	n	Experimental group			n	Control group		
		Mean (SD)	Range	95%CI		Mean (SD)	Range	95%CI
**2** **h**	42	1.2 (2.2)	0–8	0.7–2.0	42	2.3 (2.6)	0–8	1.6–3.2
**12–24** **h**	42	0.6 (1.3)	0–5	0.3–1.1	42	0.8 (1.7)	0–6	0.4–1.5
**36–48** **h**	42	0.2 (0.9)	0–5	0.1–0.8	42	0.9 (1.7)	0–6	0.5–1.5
**10–20 days**	39	0.1 (0.5)	0–3	0.0–0.5	36	1.4 (2.1)	0–7	0.8–2.2

Linear mixed-effects model p-value: group = **0.002**; stage **< 0.001**; group x stage = 0.170; SD = standard deviation; CI = confidence interval; *h* = hours.

The table shows the group's effect indicating that the mean score is lower in the experimental group, regardless of the stage, and the main stage's effect, indicating that there is a variation in the mean score of pain, with a decrease in stages 1, 2 and 3 and an increase in stage 4 for the control group. Pain intensity varied regardless of the type of intervention and time or type of intervention and the postpartum period with no group-stage interaction.

Table 2 displays the analysis of the repair time registered by a digital timer in minutes. The repair time was statistically shorter in the experimental group than in the control group (p-value < 0.001).

**Table 2:** Perineal repair time.

Group	n	Time (minutes)			p-value⁎
		Mean (SD)	Range	95%CI	
Experimental	42	6.0 (6.0)	1–35	4.7–8.7	**<0.001**
Control	42	9.7 (5.3)	2–20	8.3–11.5	

⁎ Wilcoxon-Mann-Whitney test; SD = standard deviation.

Healing was assessed using the REEDA scale. Table 3 presents the REEDA assessment results according to the analyses. At 10–20 days after birth, a healing assessment with the REEDA scale was performed only for women who attended face-to-face appointments at the hospital.

**Table 3:** Redness, Oedema, Ecchymosis, Discharge, and Approximation (REEDA) score.

Stage	n	Experimental group			n	Control group		
		Mean (SD)	Range	95%CI		Mean (SD)	Range	95%CI
**12–24** **h**	42	0.8 (1.4)	0–5	0.4–1.3	42	0.8 (1.1)	0–5	0.5–1.2
**36–48** **h**	42	0.7 (1.2)	0–6	0.4–1.2	42	0.8 (1.1)	0–4	0.5–1.2
**10–20 days**	12	0.0 (0.0)	0–0	0	12	1.0 (0.6)	0–2	0.7–1.3

Linear mixed-effects model p-value: group = 0.170; stage **<** **0.040**; group x stage = 0.054; SD = standard deviation; CI = confidence interval; *h* = hours.

The stage's effect showed statistical significance, indicating an improvement in healing throughout the stages in the experimental group. This difference may have been biased by losses of follow-up.

Women's satisfaction is shown below (Table 4).

**Table 4:** Women's satisfaction.

Stage	n	Experimental group					Control group					
		Dissatisfied	Satisfied	Very satisfied	n	Dissatisfied			Satisfied		Very satisfied	
		n (%)	n (%)	n (%)			n (%)		n (%)		n (%)	
**2** **h**	42	2 (4.8)	11 (26.1)	29 (69.0)	42			3 (7.1)		20 (47.6)		19 (45.2)
**12–24** **h**	42	1 (2.4)	5 (12.0)	36 (85.7)	42			–		16 (38.1)		26 (61.9)
**36–48** **h**	42	–	5 (12.0)	37 (88.1)	42			–		7 (16.7)		35 (83.3)
**10–20 days**	39	–	2 (5.1)	37 (94.9)	36			1 (2.8)		8 (22.2)		27 (75.0)

Generalised linear model p-value: group = **0.003**; stage **<** **0.001**; group x stage = 0.454; *h* = hours.

Regarding women's satisfaction with the perineal repair assessed through the visual analogue scale, there was statistical significance in the group's and stage's effect independently. Women allocated to the experimental group showed higher satisfaction with the intervention compared to the control group (*p* < 0.005), regardless of the stage. Women in both groups were more satisfied with the repair over time (*p* < 0.001). The percentage of very satisfied women at stages 3 and 4 was lower in the control group.

There was no statistical significance in the stage-group interaction, demonstrating that the improvement in satisfaction occurred due to the intervention and time, with no interaction between them.

In the experimental group, six women had the tears sutured according to the previously described technique due to a failure in the adhesion of the surgical glue to the tissue. Five of these cases were associated with profuse bleeding, three were identified as postpartum haemorrhage, and two were connected to profuse bleeding of the tear itself but not diagnosed as postpartum haemorrhage. One of the failures to complete the repair occurred due to the professional's difficulty in approximating the tear's edges manually while applying the surgical glue.

For the repair with surgical glue to be done correctly, it is essential that the steps of the technique described in the methods are followed correctly, so the professional must be able to approximate the edges of the tear, dry the excess blood and fluids with a gauze or compress, and then apply the glue in the smallest amount possible (application drop-by-drop is recommended). The successful adhesion of the glue seems to be related to the application on dry tissue, in a small amount, and with the edges well approximated.

The glue polymerisation occurs approximately 30 s after placing it on the tissue, and once it adheres well, it is not likely to come off. However, if it is impossible to stop the bleeding while manually approximating the edges of the tear or, in case of profuse bleeding as in postpartum haemorrhages, adherence of the surgical glue may be difficult due to the intense humidity of the site of application. Complete surgical glue detachment occurred 1 to 12 days after application, but none of the participants had the wound edges separated.

Two women had wound dehiscence during the postpartum follow-up, one in the experimental group and one in the control group. The woman in the experimental group was evaluated 6 days postpartum by the obstetrician at the hospital where this research was carried out, and minor dehiscence was observed without anatomical changes or the need for a new intervention. A topical analgesic was prescribed, and the woman was released. The control group participant was seen at the Basic Health Unit around 10 days postpartum, and according to the participant's report, a separation of the edges of the vaginal mucosa was observed. Both women were offered home visits and postpartum follow-up appointments but chose to maintain their follow-ups at the Basic Health Unit.

## 4 Discussion

In this study, using surgical glue for perineal repair resulted in less pain than using sutures. Even with the use of a local anaesthetic before suturing in the control group, the experimental group reported lower pain scores at all stages, including the first (up to 2 h after the procedure), which is a critical issue to be considered.

This finding is likely related to the shorter time of manipulation of the site to perform the repair. In the experimental group, the completion of the procedure was 3.7 min faster. A lower pain score in all stages and a shorter time of manipulation for trauma repair may have influenced women's satisfaction, which was higher in the experimental group.

Regarding the pain reported in stage 4, the difference in the score was 1.3 points on a scale of 0 – 10. However, the interval for performing this assessment was 10 – 20 days after birth; therefore, the number of days postpartum may have influenced women's responses, considering that pain decreased in both groups with passing days. Perineal pain may vary depending on the complexity of the tear and its complications. Usually, first-degree perineal tears are simple and quickly healed (American College of Obstetricians and Gynecologists' Committee on Practice Bulletins 2016). Therefore, when repairs are necessary, it is essential to consider methods that contribute to adequate healing without causing harm or increasing pain.

Only first-degree tears that needed intervention according to the evaluation of the birth attendant professionals were repaired. No routine suturing was performed, as it could have biased results.

Ochiai et al. (2020) evaluated the use of non-surgical ethyl-2-cyanoacrylate glue in perineal repair and, despite its being a non-surgical glue, the results were in line with those found in this study – women allocated to the experimental group had greater satisfaction and less pain, and the procedure time was shorter. Using surgical glue only to keep the tissue closed (the mucosa and musculature were sutured), Chamariya et al. (2016) observed pain during the procedure in 16% of the participants of the experimental group and 72% of the participants of the group that had a repair with suture, even with the use of local anaesthetic for the stitching. Similar results were found in the pilot study that preceded this clinical trial (Teixeira, 2018).

Feigenberg et al. (2014) evaluated perineal pain using surgical glue octyl-2-cyanoacrylate in first-degree lacerations on a scale from 0 to 10, as used in this study. They observed a difference of 2.42 fewer points between the repair using glue compared to suture, agreeing with other studies that found less painful sensations among women undergoing repair with surgical glue.

In the follow-up between 10 and 20 days postpartum, Marks et al. (2020) observed higher perineal pain scores among women receiving sutures with a difference of 1.36 points. Feigenberg et al. (2014) found no statistically significant difference between the procedures at 6 or more weeks postpartum. However, 86% of participants with previous experience with sutures reported preferring surgical glue.

In the literature review, despite the methodological differences among the studies, there was a general tendency of decreased perineal pain with the use of surgical glue for the repair of perineal tears or just for the closure of the skin tissue in tears and episiotomies (Marks et al., 2020; Feigenberg et al., 2014; Ochiai et al., 2020; Chamariya et al., 2016; Teixeira et al., 2020; Dasrilsyah et al., 2021; Swenson et al., 2019; Bowen and Selinger, 2002).

The data regarding perineal healing in this study should be interpreted carefully, since, due to the restrictions of the COVID-19 pandemic, it was not possible to see all women in face-to-face postpartum appointments. Data from stages 2 and 3 were collected during women's stays at the birth centre. In stage 4 of the study, there was a significant loss of follow-up. Dissatisfaction with the repair was not reported in any group.

REEDA healing scores were very low in both groups overall. The scale adds up to 15 points, with higher scores indicating worse healing. The average points and the median found in both groups did not exceed 1 point.

Marks et al. (2020)and Feigenberg et al. (2014) did not find aesthetic and functional differences between the groups evaluated 6 weeks after birth. The other studies in the area present similar results without statistical differences in the healing of both procedures (Ochiai et al., 2020; Chamariya et al., 2016; Dasrilsyah et al., 2021; Swenson et al., 2019).

It may be that the healing outcomes in this research do not directly reflect the participants' results, since there was a high loss of postpartum follow-up. However, it is unlikely that significant problems approximating the edges of the lacerations occurred in either group, as the women were followed up by telephone and were encouraged to report adverse effects of the procedures.

In our study, when repairs were performed with sutures, they remained in the tissue for a more extended period when compared to the glue, since in the evaluation of stage 4 (10 – 20 days), glue residues were not found in the experimental group, but suture material was found in the control group. Sutures seem to produce better coaptation of the edges precisely because of the approximation generated by the permanence of the thread in the tissue. However, as it degrades, the thread may have produced small failures in the coaptation that were not initially observed.

The time needed for the repairs in the pilot study was more extended than the repair time in this study. In the pilot study conducted in 2017, the average time to repair the laceration in the experimental group was 5 min, and in the control group, it was 21 min. However, the small number of participants may have caused this difference.

As in the present study, the repair time in previous studies with other glue compositions showed a shorter time for glue repair than with sutures However, the time spent to complete repairs with surgical glue in our study was longer than the others (Feigenberg et al., 2014; Ochiai et al., 2020).

This difference was likely because, in both previous studies, excessive bleeding from the perineal tears was an exclusion criterion, unlike the present study, which considered all first-degree perineal lacerations that needed repair, including those with excessive bleeding. Repair may take longer to be completed if perineal trauma has excessive bleeding, since it is necessary to dry the site constantly to apply the surgical glue. Studies comparing the application of surgical glue only to the skin layer also showed a shorter time using surgical glue than sutures (Chamariya et al., 2016; Mota et al., 2009; Rogerson et al., 2000).

### 4.1 Strengths and limitations

Of the 375 eligible women, only six refused to participate, indicating high adherence and desire to participate in the research.

The impossibility of recruiting women during pregnancy could be highlighted as a limitation, since they came from various health services scattered around the city, and it was not possible to contact them during prenatal care to obtain consent to participate in the research. Therefore, women were recruited during their stay at the birth centre.

Another significant limitation was the use of a non-validated visual analogue scale and the timing of data collection, which took place during the COVID-19 pandemic; therefore, only some women were assessed face-to-face during the follow-up appointments after hospital discharge.

It is worth considering that some wide confidence intervals may limit conclusions about the best evidence on the perineal repair method. However, these results may help design non-inferiority trials to study the repair of perineal tears with several types of surgical glue.

This study presents the feasibility of using surgical glue to repair first-degree perineal trauma. The surgical glues available in Brazil's market are currently imported and, according to currency exchange rates, have a high cost and significant price fluctuation. So, the cost-effectiveness of using imported glue should be studied.

## 5 Conclusions

The surgical glue in this study was a favorable alternative to suture repair for first-degree perineal lacerations, as it resulted in less perineal pain, faster repair, and greater satisfaction than perineal sutures after birth. The healing process was similar in both cases.

## Funding sources

No external funding.

## CRediT authorship contribution statement

**Thaís Trevisan Teixeira:** Conceptualization, Methodology, Writing – original draft, Data curation, Writing – review & editing. **Maria Luiza Riesco:** Data curation, Visualization, Investigation, Supervision, Software, Validation, Writing – review & editing.

## Declaration of Competing Interest

The authors declare that they have no known competing financial interests or personal relationships that could have appeared to influence the work reported in this paper.

## Appendix Supplementary materials

Image, application 1

Image, application 2
