# Supplementary material for: Surgical adhesive glue to repair first-degree perineal tears in vaginal birth: A randomised controlled clinical trial
Source: Int J Nurs Stud Adv. 2023 May 12;5:100130. doi: 10.1016/j.ijnsa.2023.100130 (PMC11080570; doi:10.1016/j.ijnsa.2023.100130)
Supplement: Supplementary file 1 [file mmc1.docx]

Table – Women’s characteristics

| **Characteristic** | **EG (n = 42)** | **CG (n = 42)** |
| --- | --- | --- |
|  | **n (%)** | **n (%)** |
| **Skin colour** |  |  |
| Brown | 28 (66.7) | 20 (47.6) |
| White | 9 (21.4) | 11 (26.2) |
| Black | 5 (11.9) | 11 (26.2) |
| **Education level** |  |  |
| Primary - incomplete | 6 (14.3) | 2 (4.8) |
| Primary - complete | 13 (30.9) | 14 (33.3) |
| Secondary - complete | 23 (54.8) | 26 (61.9) |
| **Occupation** |  |  |
| Paid | 23 (54.8) | 18 (42.9) |
| Not paid | 16 (38.1) | 20 (47.6) |
| Student | 3 (7.1) | 4 (9.5) |
| **Marital status** |  |  |
| Live with partner | 33 (78.6) | 29 (69.0) |
| Not live with partner | 9 (21.4) | 13 (31.0) |
| **Previously sutured perineal trauma** |  |  |
| Yes | 19 (45.2) | 17 (40.5) |
| No | 23 (54.8) | 25 (59.5) |
| **Companion during birth** |  |  |
| Yes | 39 (92.9) | 41 (97.6) |
| No | 3 (7.1) | 1 (2.4) |
| **Intrapartum antibiotic** |  |  |
| Yes | 2 (4,8) | 4 (9,5) |
| No | 40 (95,2) | 38 (90,5) |
|  |  |  |
|  | **Mean (s.d.) [range]** | **Mean (s.d.) [range]** |
| **Age** (years) | 25,0 (6,8) [16-41] | 25,0 (6,0) [16-41] |
| **Gestational age** (weeks) | 38,9 (1,3) [36-41] | 39,0 (1,4) [35-41] |
| **Body mass index** | 29,8 (4,4) [19,8-39,6] | 29,0 (4,5) [17,6-37,0] |
| **Previous birth** | 0,8 (0,8) [0-2] | 0,7 (1,0) [0-4] |
| **Time between admission-birth** (h) | 10,7 (12,1) [0,9-70,7] | 8,9 (9,1) [0,7-42,1] |
| **Time between aminorexes-birth** (h) | 5,7 (11,7) [0-73] | 6,2 (9,0) [0-48] |
| **Newborn weight** (g) | 3078 (431) [2010-3900] | 3226 (440) [2125-3960] |

EG = experimental group; CG = control group; ^a^Chi-square test; ^b^Wilcoxon-Mann-Whitney test; ^c^Student-t test
