# Supplementary material for: Surgical adhesive glue to repair first-degree perineal tears in vaginal birth: A randomised controlled clinical trial
Source: Int J Nurs Stud Adv. 2023 May 12;5:100130. doi: 10.1016/j.ijnsa.2023.100130 (PMC11080570; doi:10.1016/j.ijnsa.2023.100130)
Supplement: Supplementary file 2 [file mmc2.pdf]

IJNS AUTHOR CHECKLIST ***Authors of all papers should submit this checklist plus the checklist from the relevant reporting guideline together with their manuscript.*** *Part 1* identifies basic requirements for the manuscript submission *(mandatory for all submissions)*

Part 2 identifies recognized guidelines for scientific reporting, which you should use to prepare your manuscript *(required for systematic reviews and original research)*

PART 1 Basic requirements	***Author response or further detail – please complete the boxes below***
Word count	6087 words
Was ethical approval given and by whom? (give any reference number)	The study was approved by the Research Ethics Committees of the School of Nursing of the University of São Paulo with approval number 3.890.595 on February 28, 2020, by the Instituto Israelita Albert Einstein (administrators of the Municipal Hospital) with approval number 3.957.461 on April 6, 2020, and by the Municipal Health Department of Sao Paulo with approval number 4.191.287 on August 4, 2020.
Please state any conflicts of interest	None.
Please state sources of funding and the role of funders in the conduct of the research	No external funding.
Please state any study registry number (e.g. ISRCTN)	Registered on The Brazilian Clinical Trials Registry number RBR-52y5tq (http://www.ensaiosclinicos.gov.br/rg/RBR-52y5tq/), on July 16, 2020.

For the items below, please tick in the right hand column to confirm you have included/addressed the items:		**Tick**
Title	Confirm that the title is in the format 'Topic / question: design/type of paper' and identifies the population / care setting studied. *(e.g. The effectiveness of telephone support for adolescents with insulin dependent diabetes: controlled before and after study).* The structure is optional for discussion papers, editorials and letters)	ok
Abstract	A structured abstract appropriate to the design of the study is included (see *guidelines for authors)*.	ok
	No references are cited in the abstract.	ok
Key words	Between four and ten key words have been provided in alphabetical order, which accurately identify the paper's subject, purpose, method and focus. Use the Medical Subject Headings (MeSH®) thesaurus or Cumulative Index to Nursing and Allied Health (CINAHL) headings where possible (see http://www.nlm.nih.gov/mesh/meshhome.html).	ok
Highlights	Bullet points have been included that identify existing research knowledge relating to the specific research question / topic (what is already known about the topic?) and a summary of the new knowledge added by this study (what this paper adds) *(see Guide for Authors*, does not apply to editorials or letters)	ok
Abbreviations	No abbreviations are used in the title / abstract. Use of abbreviations /acronyms in the paper is minimised and restricted to those that are likely to be universally recognized (e.g. USA)	ok
References	All citations in the paper have a complete and accurate reference in the reference list (see *Guide for Authors)*	ok
Other Published accounts	*All* published and in press accounts of the study from which data in this paper originate are referred to in the paper and the relationship between this and other publications from the same study is made clear (see *Guide for Authors*) (Please upload copies of all previous, current and under review publications from this study and / or give full details below)	ok

	Please provide references of ANY other papers using data from the study that this paper is based on) below.	ok
	The study is referred to by a distinctive name which will be used in any future publications to identify that it is the same study (e.g. RN4Cast)	ok
Authorship	All authors and contributors sufficiently acknowledged as per Guide for Authors.	ok

PART 2 Standards of reporting	The editors require that manuscripts adhere to recognized reporting guidelines relevant to the research design used. These identify matters that should be addressed in your paper. Authors of research papers and systematic reviews are required to submit a checklist relevant to the research design they have used. The checklist will be drawn on within the peer review process. Please indicate which guideline (below) that you have referred to and ensure that the relevant checklist is uploaded.<br><br>These are not quality assessment frameworks and your study need not meet all the criteria implied in the reporting guideline to be worthy of publication in the IJNS. The checklists do, however, identify essential matters that should be considered and reported upon. For example, a controlled trial may or may not be blinded but it is important that the paper identifies whether or not participants, clinicians, outcome assessors and analysts were aware of treatment assignments.<br><br>*Reporting guidelines endorsed by the IJNS are listed below:*	**Checklist submitted****
Observational cohort, case control and cross sectional studies	STROBE **St**rengthening the **R**eporting of **Ob**servational Studies in **E**pidemiology<br>http://www.equator-network.org/index.aspx?o=1032	
Quasi experimental / non-randomized evaluations	TREND - Transparent Reporting of Evaluations with Non-randomized Designs<br>http://www.cdc.gov/trendstatement/	
Randomised (and quasi-randomised) controlled trial	CONSORT – Consolidated Standards of Reporting Trials<br>http://www.equator-network.org/index.aspx?o=1032	ok
Study of Diagnostic accuracy / assessment scale	STARD Standards for the Reporting of Diagnostic Accuracy studies<br>http://www.equator-network.org/index.aspx?o=1032	
Systematic Review of Controlled Trials	PRISMA - Preferred Reporting Items for Systematic Reviews and Meta-Analyses<br>http://www.equator-network.org/index.aspx?o=1032	
Systematic Review of Observational Studies	MOOSE Meta-analysis of Observational Studies in Epidemiology<br>http://www.equator-network.org/index.aspx?o=1032	
Qualitative studies	COREQ: Consolidated criteria for reporting qualitative research Tong, A., Sainsbury, P., Craig, J., 2007. Consolidated criteria for reporting qualitative research (COREQ): a 32-item checklist for interviews and focus groups. *International Journal for Quality in Health Care* 19 (6), 349-357. (http://dx.doi.org/10.1093/intqhc/mzm042)	
Other (please give source)		
Not applicable (please elaborate)		
